# Stimulus-Related Independent Component and Voxel-Wise Analysis of Human Brain Activity during Free Viewing of a Feature Film

**DOI:** 10.1371/journal.pone.0035215

**Published:** 2012-04-05

**Authors:** Juha M. Lahnakoski, Juha Salmi, Iiro P. Jääskeläinen, Jouko Lampinen, Enrico Glerean, Pia Tikka, Mikko Sams

**Affiliations:** 1 Brain and Mind Laboratory, Department of Biomedical Engineering and Computational Science (BECS), School of Science, Aalto University, Espoo, Finland; 2 Advanced Magnetic Imaging (AMI) Centre, School of Science, Aalto University, Espoo, Finland; 3 Department of Motion Picture, Television and Production Design, School of Arts, Design and Architecture, Aalto University, Helsinki, Finland; Macquarie University, Australia

## Abstract

Understanding how the brain processes stimuli in a rich natural environment is a fundamental goal of neuroscience. Here, we showed a feature film to 10 healthy volunteers during functional magnetic resonance imaging (fMRI) of hemodynamic brain activity. We then annotated auditory and visual features of the motion picture to inform analysis of the hemodynamic data. The annotations were fitted to both voxel-wise data and brain network time courses extracted by independent component analysis (ICA). Auditory annotations correlated with two independent components (IC) disclosing two functional networks, one responding to variety of auditory stimulation and another responding preferentially to speech but parts of the network also responding to non-verbal communication. Visual feature annotations correlated with four ICs delineating visual areas according to their sensitivity to different visual stimulus features. In comparison, a separate voxel-wise general linear model based analysis disclosed brain areas preferentially responding to sound energy, speech, music, visual contrast edges, body motion and hand motion which largely overlapped the results revealed by ICA. Differences between the results of IC- and voxel-based analyses demonstrate that thorough analysis of voxel time courses is important for understanding the activity of specific sub-areas of the functional networks, while ICA is a valuable tool for revealing novel information about functional connectivity which need not be explained by the predefined model. Our results encourage the use of naturalistic stimuli and tasks in cognitive neuroimaging to study how the brain processes stimuli in rich natural environments.

## Introduction

Understanding how the human brain processes information in complex everyday situations presents one of the ultimate challenges in cognitive neuroscience. The vast majority of previous neuroimaging experiments have strived to increase our understanding of the neural basis of perception using carefully controlled stimuli and tasks. However, there are reports showing that results obtained under such settings do not always generalize to real life conditions. For instance, a recent study by David and co-workers [1] demonstrated differences in the spectro-temporal tuning properties of the auditory cortex with natural speech *vs.* artificial sound stimuli. Schultz and Pilz [2] showed that dynamic facial expressions cause higher activity than static ones even in the face sensitive areas. Moreover, using more natural stimuli assists in observing patterns of brain activation that are difficult to observe using simple stimuli. For example, Bartels and Zeki [3] demonstrated that distinct brain areas exhibit more independent activity patterns while subjects are watching natural movies when compared to results obtained using short video clips and a blocked paradigm. However, what are currently lacking, and what has deterred neuroimaging studies using naturalistic stimuli, are well-established methods that allow analysis of the multidimensional brain responses to the features of the highly complex naturalistic stimulus. Here, our goal was to develop models and compare tools that enable one to study the human brain under ecologically valid naturalistic stimulus and task conditions.

In recent studies, hemodynamic brain responses during naturalistic stimulation, such as feature films or normal connected speech, have been shown to be amenable to study using a relatively simple model-free voxel-wise inter-subject correlation (ISC) analysis [4], [5], [6] (for a review see [7]). Another model-free method, independent component analysis (ICA), relies on the separation of maximally independent components of brain activation without information of external stimulation by minimizing the mutual information [8] of source estimates called independent components (ICs). The classical example of ICA is the cocktail party problem, where speech from multiple sources is separated from observed mixed signals without prior knowledge of the speakers. In fMRI studies, ICA has been used to extract similarly activated networks of brain regions during rest and during natural viewing [3], [5], [9].

Here, we hypothesized that by annotating quantitatively the movie stimulus, it is possible to disclose the specificity of IC-based networks to various stimulus features in the movie. In fact, in recent pioneering studies, stimulus-modeling approaches such as quantification of local and global movement over time in a movie [10] or quantification of sound features [11] have been successfully implemented, thus suggesting that development of more advanced stimulus annotation methods helps the analysis of brain activity during natural audiovisual stimulation, particularly when stimuli are not pre-selected and multitude of stimulus features are overlapping. However, as the number of overlapping features increases there is a chance that the explanatory variables become linearly dependent rendering traditional general linear models (GLM) without a unique solution.

In the current study, our aim was to critically test whether annotating several robust visual and auditory features that are known to activate multiple brain networks makes it possible to detect such networks during a naturalistic free viewing condition and to compare the results of the model-free ICA approach to those obtained using a conventional voxel-wise GLM analysis. A feature film “Match Factory Girl” (dir. Aki Kaurismäki, 1990) was re-edited in order to shorten the story to allow larger part of the main story line to be shown to healthy volunteers during fMRI scanning. From the movie, we extracted eight auditory features (zero crossing rate, spectral spread, entropy, root mean square (RMS) energy, speech, music, lead singing, and background singing), both automatically and manually. We also extracted seven visual features (contrast edges and six motion categories: hand, body, head, mechanical, large scale, and inferred). The motion categories were manually scored according to the perceived strength of motion. The spatial location of the main characters of the movie and their heads and hands and non-biological moving objects were also annotated using in-house developed semi-automated motion recognition software. Temporal dynamics of the extracted features were then compared with activation time courses of ICs and with single voxels.

We hypothesized that to successfully reveal brain areas participating in coding of a complex natural stimulus we would need a complex stimulus model with a rich collection of features. We expected that such models could be used to explore brain areas and networks of areas involved in processing complex stimulus feature combinations, while any single feature alone would not be sufficient to explain the activity of the network. We selected six ICs of interest using temporal inter-subject correlation of IC time courses and employed GLM approach to fit the collections of stimulus annotations. Permutation testing was utilized to correct the increased risk of over fitting. The weights of each feature in the IC and voxel-wise models were used to inspect the extent to which annotated features are encoded at each network, and were further compared with voxel-wise analysis using isolated stimulus features.

## Materials and Methods

### Subjects

Twelve healthy native speakers of Finnish were studied. Two of the subjects were excluded from the study due to technical problems leaving ten subjects (22–43 years, mean 31; two female; two left-handed) included in the final analysis. Permission for the study was acquired from the ethical committee of Hospital district of Helsinki and Uusimaa. The study was carried out in accordance with the guidelines of the declaration of Helsinki, and written informed consent was obtained from each subject prior to participation.

### Stimuli and Procedure

The subjects watched 22 minutes and 58 seconds of a Finnish language film in the fMRI scanner. The feature film “Match Factory Girl” (dir. Aki Kaurismäki, 1990, original length 68 min) was re-edited (by a professional movie director and co-author PT*) in order to adapt the story to be imaged within the constraint of 20000 slice acquisitions allowed by the MRI scanner. The shorter version retained the main storyline and smooth flow of scenes. Subjects were instructed to avoid any movements and watch the movie during fMRI scanning. The beginning of the movie was synchronized to the beginning of the scanning using Presentation software (Neurobehavioral Systems Inc., Albany, CA, USA). The movie was back-projected on a semitransparent screen using a 3-micromirror data projector (Christie X3, Christie Digital Systems Ltd., Mönchengladbach, Germany). The subjects viewed the screen at 34 cm viewing distance *via* a mirror located above their eyes. Projected image width was 19.7 cm. The audio track of the movie was played to the subjects with an UNIDES ADU2a audio system (Unides Design, Helsinki, Finland) via plastic tubes through porous EAR-tip (Etymotic Research, ER3, IL, USA) earplugs. The intensity of the auditory stimulation was selected to be loud enough to be heard over the scanner noise and was kept constant for all participants.

### Functional Magnetic Resonance Imaging

Functional brain imaging was carried out with a 3.0 T GE Signa Excite MRI scanner (GE Medical Systems, USA) using a quadrature 8-channel head coil. The imaging area consisted of 29 functional gradient-echo planar (EPI) oblique slices (thickness 4 mm, between-slices gap 1 mm, in-plane resolution 3.4 mm × 3.4 mm, voxel matrix 64 × 64, TE 32 ms, TR 2 s, flip angle 90°). Images were acquired continuously during the experiment. In addition, a T1-weighted spoiled gradient echo volume was acquired for anatomical alignment (SPGR pulse sequence, TE 1.9 ms, TR 9 ms, flip angle 15°). The T1 image in-plane resolution was 1 mm × 1 mm, matrix 256 × 256 and slice thickness 1 mm with no gap. Each dataset consisted of 689 functional volumes.

### Preprocessing

All the preprocessing steps were performed using FSL [12], [13]. Motion correction was applied using MCFLIRT [14], and non-brain matter was removed using BET [15]. Values for intensity threshold and threshold gradient in BET were searched manually by changing the parameters and visually inspecting each brain extracted volume until the results were satisfactory. The datasets were registered to 2 mm MNI152 standard space template using the brain extracted T1 weighted image of each individual subject as an intermediate step using FLIRT [14]. Registration from functional to anatomical volumes was done using 12 degrees of freedom (DOF). Anatomical images were registered to the standard template using 7 DOF allowing translation, rotation, and global scaling. Volume data were smoothed using a Gaussian kernel with full width at half maximum (FWHM) of 6.0 mm. High-pass temporal filtering was applied using Gaussian-weighted least-squares straight line fitting, with sigma 100 s, with the first 10 volumes of each dataset discarded (blank screen was presented during these volumes).

### Data Analysis

Two approaches were used for analysis of the functional data: model free ICA and model driven GLM analysis. ICA searches for unknown latent signals in the data through the general assumption of statistical independence by minimizing or maximizing an objective function such as mutual information, negentropy [16], or joint entropy of source estimates, as used by the Infomax algorithm used in the current study [17]. Thus, ICA reveals the functional connectivity structure of the brain independent of external stimulation. Time courses of ICs in spatial ICA are calculated as the weighted average of the voxel time courses and do not represent the true activity of each voxel equally well but are typically dominated by the strongest voxels in each IC. In contrast, GLM relies only on the parameters of the model, such as traditional model of the experimental design, subject ratings, annotations of the stimulus, and the model of the hemodynamic response to predict the activity of individual voxels.

Group ICA was performed using GIFT (Group ICA fMRI Toolbox, http://icatb.sourceforge.net/). Infomax was chosen as the ICA algorithm with default parameters. ICA was performed 100 times with random initialization and bootstrapping enabled using ICASSO package included in GIFT. The IC clustering is based on the absolute value of the spatial correlation coefficient, and is described in detail in ICASSO publications [16], [18]. Because the number of ICs (90) estimated by the minimum description length approach yielded very unstable ICs, the number of ICs was selected *post hoc* by repeating the bootstrap calculation with several dimensionalities. The most stable results were found when the data dimensionality was 40, which was selected for the final analysis. ICs that were found in all 100 repetitions and the average intra-cluster similarity of which was >0.9 were sorted according to the mean pair-wise temporal correlation coefficient across subjects. Fisher’s Z transform was applied to correlation coefficients before calculating the mean. ICs for which the mean pair-wise correlation was significant at p < 0.001, uncorrected, were selected for subsequent analysis, and are presented in the results.

Collections of annotation time courses (see below) were fitted to IC time courses using GLM fitting in MATLAB (Mathworks, Natick, MA, USA). Finally, similar GLM fitting was performed also for each voxel’s time course separately. Voxel-wise analysis was done separately on single subject data and mean activity across subjects. The mean time courses were calculated by standardizing the subjects’ voxel time courses to zero mean and normalizing the variance to unity prior to calculating the mean across subjects for each voxel. The correlation coefficients for IC and voxel-wise time courses were calculated with the fitted sum of annotations for that IC or voxel.

Sensitivity of ICs to auditory features was compared by calculating the weights in GLM for single auditory features in isolation for each subjects IC time course. We performed paired t-tests to compare for which features the weights differed between ICs. T-tests were used to test which weights differed significantly from zero across subjects.

Significance threshold level for correlation coefficients of the GLM fitted annotation time courses was estimated using permutation testing. The algorithm applied a circular shift to the fMRI time courses and GLM fitting of the annotations was performed on the shifted time courses. The correlation coefficients for the fitted sums of annotations and the shifted time courses were calculated. All permutations (679 different shifts) were calculated for all IC time courses (27,160 realizations) and all mean brain voxels (155,139,957 realizations). Separate thresholds were calculated for the visual and auditory models to account for over-fitting for each model individually. The probability distribution was estimated by creating a lookup table through dividing the observed correlation coefficients into 100 bins. The probability for each bin was estimated as the observed frequency of realizations and assigned to the center of the bin. More precise estimate was obtained through linear interpolation between the bins. The thresholds of significant (p < 0.001, uncorrected) correlation in the permutation test for IC time courses were r > 0.4772 for auditory model and r > 0.4230 for visual model. For voxel-wise data threshold was r > 0.4706 for auditory and r > 0.4144 for visual model. Similar permutation testing was done for the single annotations except without GLM fitting, but because of computational expense only half of the permutation distribution was sampled (i.e., circular shifting was done in two step increments). For visualization a single threshold was calculated for all auditory and visual annotations as the mean of the individual annotation thresholds for the given modality. Since we aimed to compare the results of ICA and voxel-wise analysis, we did not perform explicit multiple comparisons correction between the models in our analyses to avoid overestimating the differences of the two methods. However, areas which were not significant in the tests for the full auditory and visual models are not reported in the single feature analyses.

In the results we present only the ICs for which a) temporal correlation between subjects was significant (p < 0.001, uncorrected), b) time course of the IC was significantly correlated (p < 0.001, uncorrected) either with the auditory or visual stimulus model, and c) the IC was stable in the bootstrap test performed with ICASSO.

The overlapping *vs.* non-overlapping clusters of voxels indicated as significantly activated by ICs and GLM were extracted using MATLAB and SPM8 connected component labeling function that enforced 18 connectivity criterion (http://www.fil.ion.ucl.ac.uk/spm/software/spm8/). We selected only clusters larger than 125 voxels for the final analysis. Significance of functional connectivity between the mean time courses of the clusters was assessed with permutation testing. We performed ten million permutations to obtain a reliable estimate of the permutation distribution. Each permutation consisted of randomly selecting a seed region, and shifting its time course by at least five samples. We then calculated the correlation of the shifted time course with the time courses of all other clusters and saved the maximum value. The threshold of significant (p < 0.001, uncorrected) functional connectivity in the permutation test was r = 0.4121.

### Stimulus Annotations

**Figure 1:**
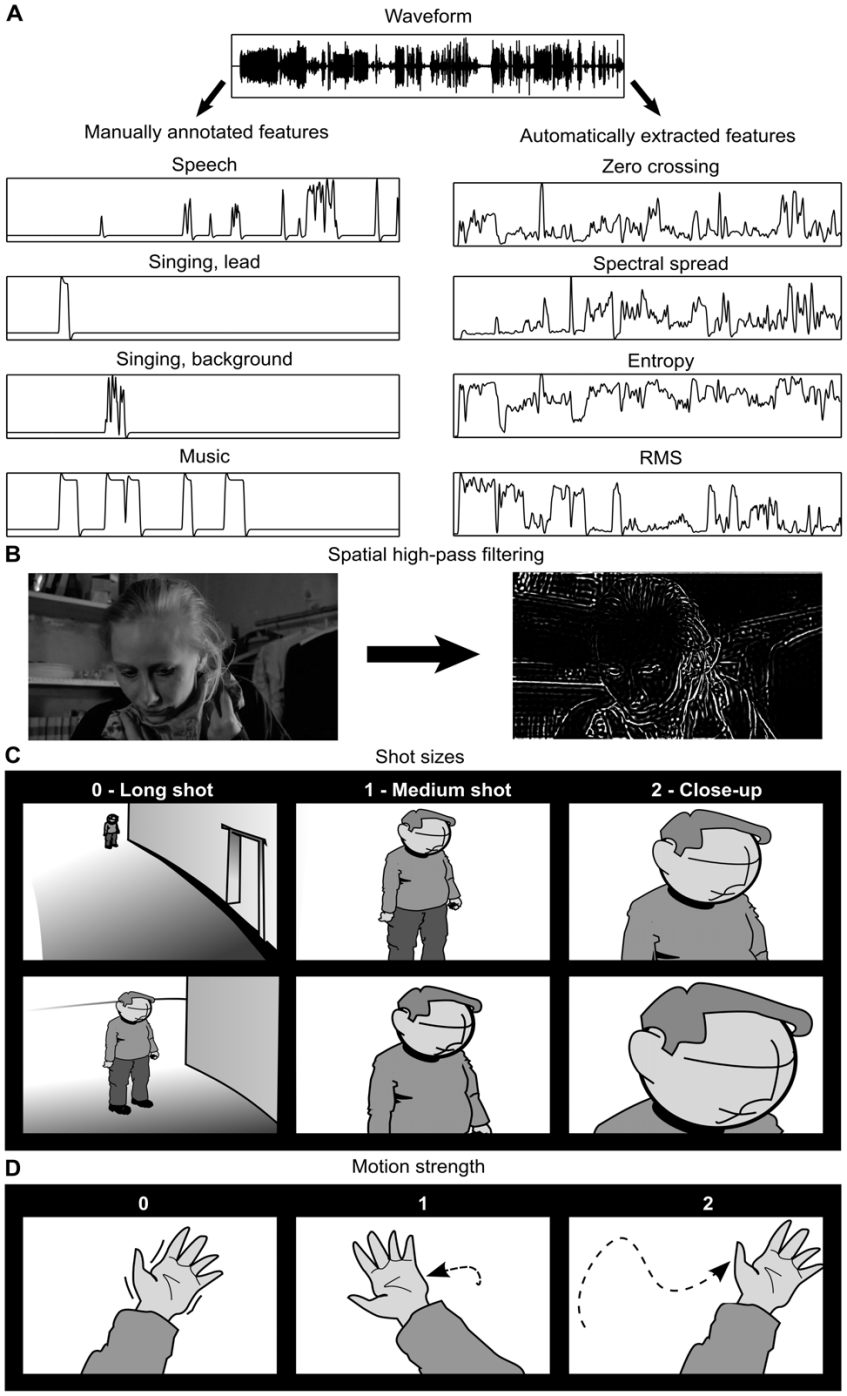
Annotation of visual and auditory features of a film. A: The presence of speech, lead singing, background singing, and music were annotated manually from the soundtrack. The zero crossing rate, spectral spread, entropy, and RMS energy sound features were extracted automatically. B: Spatial high-pass filtering was used to extract high spatial frequencies from the image to quantify to overall complexity of the image. For printing the contrast of the high-pass filtered image was increased to make the features visible. (Still images courtesy of Aki Kaurismäki and Sputnik Oy.) C: Scoring of size of body parts/objects followed the shot size convention used in cinema (long shots  =  0, medium/medium close-up shots  =  1, and close-up shots  =  2). D: Extent of motion was scored on three-step scale (no motion  =  0, intermediate motion  =  1, large motion  =  2). The overall motion score was calculated as the sum of the scores of shot size and motion strength for those time points where motion was present.

Time intervals of the movie containing speech, instrumental music, lead singing, and background singing were annotated from the sound track manually and modeled as boxcar functions with 1-s resolution. MIRToolbox [19] was used to extract a large collection of acoustic features from the sound track. We then selected the maximally independent annotations with a clear physical interpretation leaving us with the following features: zero crossing rate, spectral spread, entropy, and RMS energy (see Figure 1A). Zero crossing rate is a simple measure of noisiness, spectral spread is the standard deviation of the spectrum, and entropy describes the randomness of the spectrum. Because the entropy of silence is not defined we substituted the empty samples in the beginning of the entropy time course with values equal to the entropy of the most silent part of the sound track. All sound features were calculated in 1-s windows. All annotations were convoluted with a double gamma canonical hemodynamic response function (HRF) with a six second lag.

The video was high-pass filtered using discrete cosine transform and applying a two dimensional spatial mask with cutoff of 2π/16 rad/pixel. Cutoff frequency was selected by visual inspection of the high-pass filtered images so that only sharp contrast edges were retained in the image. Absolute values of the pixel intensities were calculated for each frame resulting in an image where the high-frequency content corresponded to high pixel intensity (Figure 1 B). Mean pixel intensity of the resulting images was calculated to quantify the sharp contrast edges in each image.

Six motion categories were annotated manually: 1) mechanical motion, 2) hand movements, 3) head movements, 4) body movements, 5) global motion (*e.g.*, camera or background or large part of the background moving), and 6) inferred motion of body parts not directly visible in the picture. Motion was scored on a scale 0–4 in a two-step process (see Figure 1). Size of the moving object in the visual field was evaluated on a scale 0–2 according to the shot size (see Figure 1 C). Motion strength was rated on a scale 0–2: 0  =  no motion, or barely visible motion, 1  =  intermediate motion, 2  =  strong motion across the view (Figure 1 D). The overall strength of perceived motion for those time windows where motion score was greater than zero was obtained by calculating the sum of size and the motion of the object. If the object was not in motion, it received a score of 0 regardless of its size on the screen.

Semi-automatic motion annotation was performed using in-house software. The object to be tracked was selected from the movie by drawing a rectangle around it. Algorithm employing Gabor filters was used to find robust landmarks within the rectangle. The object was then automatically tracked until the scene changed or the user indicated that the object is no longer in the picture. The area of the rectangle surrounding the objects was used to approximate the size of the object. The difference of the position of the centroid between frames was used to calculate the speed of the objects.

Objects included people (whole persons, their heads and hands) and non-biological objects. Large crowds of people were annotated as a single object. The time courses of area and speed were mapped to a scale similar to the manual annotations and grouped to categories (body, head, hand and mechanical). Area whose size was less than 1/10 of the whole image area received the score 0, for areas between 1/10 and 1/6 the score was 1, and larger objects received the score 2. Speed of each object was calculated as the sum of instantaneous speeds within each 1 second window, and thresholded so that speed of less than 15 pixels/second received the score 0, speeds between 15 and 50 pixels/second (corresponding approximately to 0.7 and 2.3 degrees/second in the visual field) received the score 1, and speeds faster than 50 pixels/second received the score 2. Maximum value of objects within each category was used as the measure of speed and area within each temporal window and motion time course for each category was calculated as a sum of the speed and the area similar to the manual annotations.

**Figure 2:**
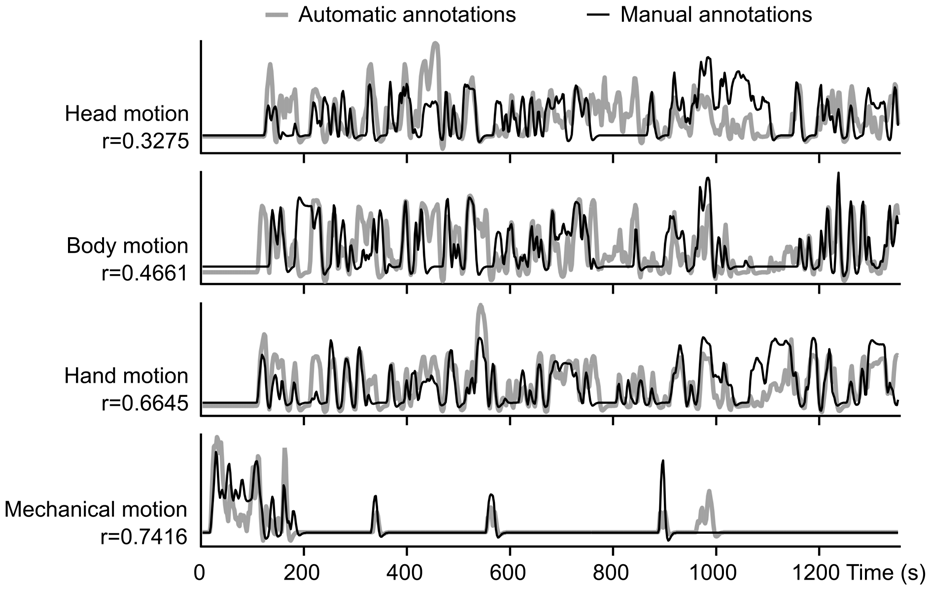
Comparison of automatic and manual annotations of four different motion categories. Correlations (r) between automatic and manual annotations are indicated on the left.

Figure 2 compares the manually and semi-automatically created models of visual motion convolved with canonical HRF. The majority of peaks in the time courses coincide in both models and the correlation between models is significant (p < 0.001) for all motion categories. However, the manual annotation of head motion differs strongly from the automatic annotations in parts of the movie, and other categories also contain sequences where the two methods yield different evaluations of the motion strength. Despite the differences both models revealed very similar spatial maps of correlated activity. The manual annotations fit the observed activity better than the semi-automatic annotations for hand and mechanical motion whereas head and body motion showed slightly higher correlations in motion sensitive areas for the automatic annotations. Correlation values for peak voxel for the manually annotated features were r = 0.3311 for head, r = 0.3967 for body, r = 0.6890 for hand, and r = 0.3641 for mechanical motion. Correlations for peak voxel and the semi-automatic annotations were r = 0.3483 for head, r = 0.4041 for body, r = 0.5682 for hand, and r = 0.3518 for mechanical motion.

**Figure 3:**
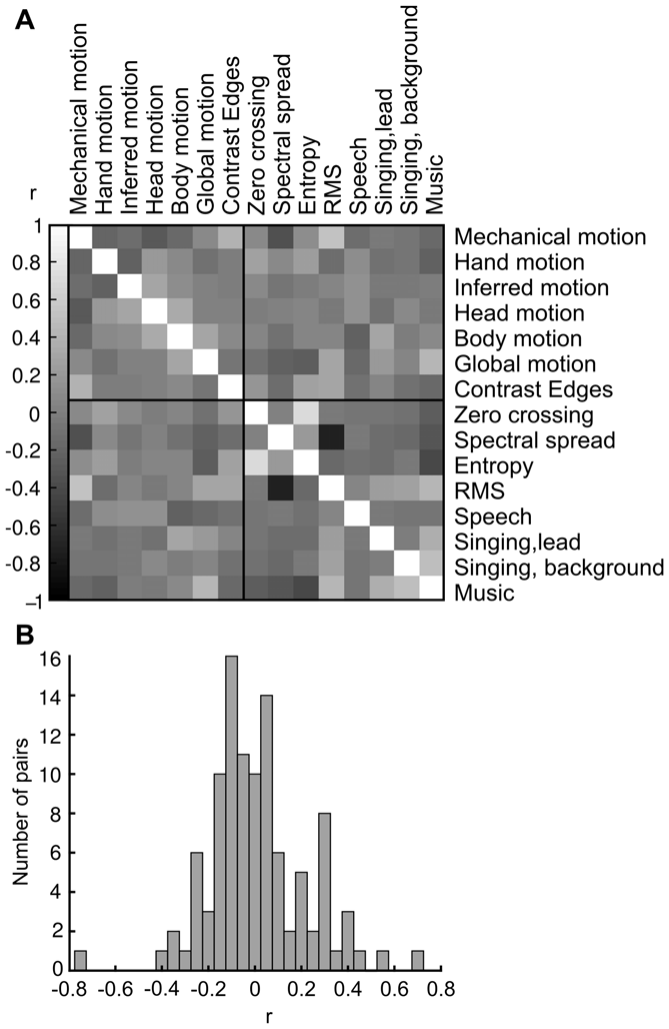
Pair-wise correlations of annotated stimulus features. A: Correlation matrix of all auditory and visual features. Vertical bar on the left shows the grey-scale code of the correlation coefficients. B: Histogram showing the distribution of the coefficients.

Figure 3 shows the pair-wise correlations of the visual and auditory features (A) and a histogram of pair-wise correlation values (B). Most feature pairs show little correlation. Strongest positive correlation is 0.70 between entropy and zero crossing rate of sound. Strongest negative correlation is -0.75 between RMS energy and spectral spread. Because high correlations between features included in the same model may cause misleading results in multiple regression methods we performed analyses for both the full models and single features, since orthogonalization of the models was not feasible in the current context.

## Results

### Auditory Independent Components

**Figure 4:**
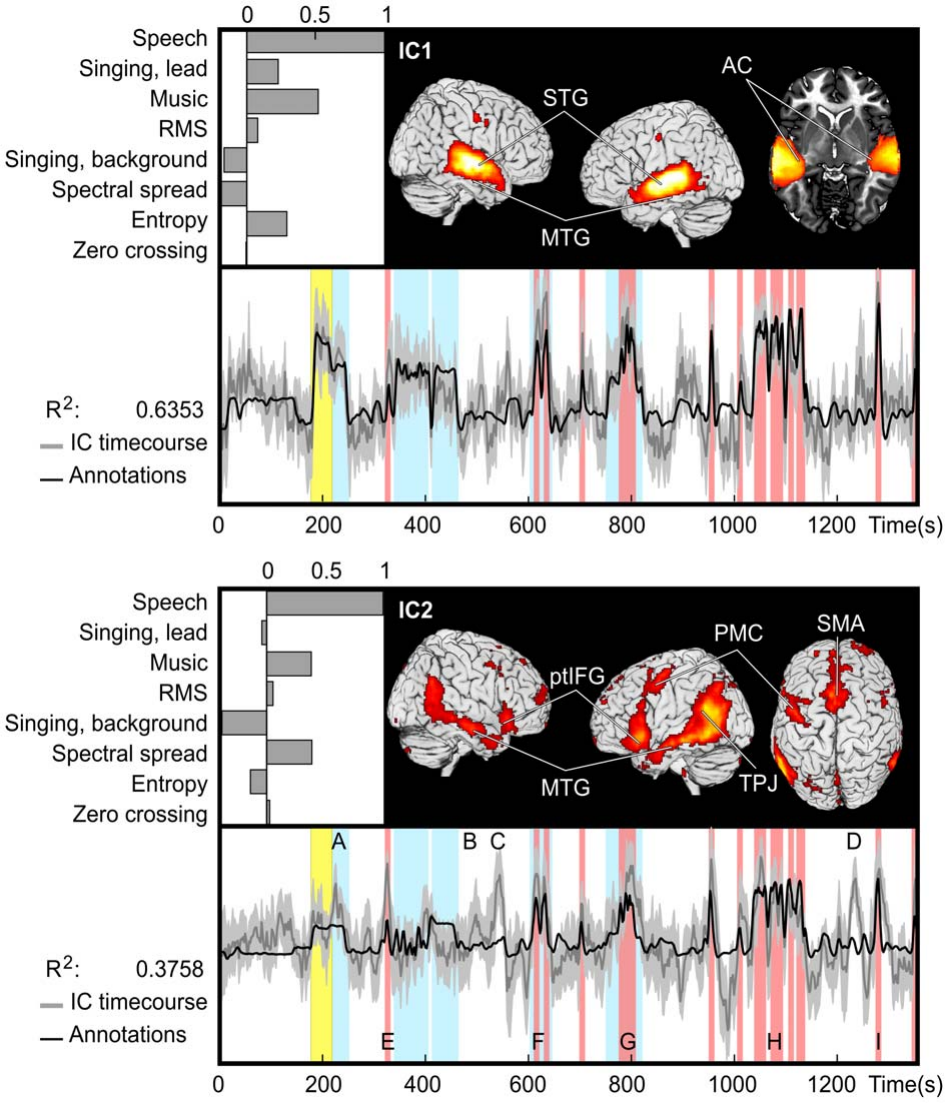
The two ICs that were found to be sensitive to auditory features in the movie. IC1 (top) encompassed bilaterally the auditory cortex (AC), superior temporal gyrus (STG), middle temporal gyrus (MTG), and relatively small activation foci in or in the vicinity of the lip representation in primary motor cortex. The normalized weights used to fit the auditory feature model to IC activity are shown in the upper left corner. The time course of the fitted stimulus model (black), mean time course (dark gray), and 95% confidence interval (light gray) of the IC are overlaid below. R^2^ indicates the coefficient of determination of the stimulus model and IC’s temporal behavior. Vertical bars show time intervals when there is speech (red), singing (yellow), and music (blue) in the sound track. IC2 (bottom) includes the MTG and inferior frontal gyrus/pars triangularis (ptIFG) in both hemispheres as well as the left temporoparietal junction (TPJ), left premotor cortex (PMC) anterior to the motor cortex cluster of IC1, and the supplementary motor area bilaterally (SMA). A–D indicate examples of instances at which activation is not explained by the auditory model while E–I highlight moments containing speech and show peaks in brain activity. Activity patterns during these instances are shown in Figure 6.

Figure 4 depicts the two ICs activating similarly across subjects that correlated with auditory features. IC1 (upper panel) is located bilaterally in the superior temporal lobe, including Heschl’s gyri, planum temporale, superior temporal gyrus (STG) and extending through superior temporal sulcus (STS) to superior parts of the middle temporal gyrus (MTG). IC1 also includes a small bilateral cluster in the precentral gyrus approximately at the lip representation area [20]. The bar graph shows the weights used in the fitting process. Weights, normalized so that the main explanatory variable receives the weight 1, reflect the sensitivity of the IC to these features. IC1 is most sensitive to speech, but is also sensitive to music. The other features that receive relatively high weights are lead (but not background) singing, and entropy. Activation time course of IC1 follows smoothly that of the model of the auditory stimulus (convoluted with canonical hemodynamic response function), the fit (R^2^) between the two being 0.6353 (p < 0.001). Mean inter-subject correlation (ISC) of the activation time courses of individual subjects (ISC ± variance) was 0.41 ± 0.02 (p < 0.001).

IC2 encompasses a widespread bilateral network of brain areas including the MTG, posterior regions of temporal lobe and temporoparietal junction (TPJ), pars triangularis region of inferior frontal gyrus (ptIFG), dorsomedial prefrontal cortex (dmPFC), premotor cortex (PMC) just anterior to the approximate location of the lip area of the motor strip, part of precuneus (Pcu), and a small cluster in the paracingulate gyrus (pCG). The clusters of cortical activity appear larger in the left hemisphere. The IC2 encompasses a more extensive network of brain areas than IC1 and, further, the areas included in the network of IC2 are ones that are typically associated with hierarchically higher-order auditory processing of than those of IC1, for example meanings of words and sentences [21], [22]. IC2 shares with IC1 its sensitivity to speech, and music, however, it appears not to be sensitive to singing. IC2 also appears to be sensitive to spectral spread, unlike IC1. Fit of the activation time courses of IC2 and auditory stimulus model is R^2^  =  0.3758 (p < 0.001). Mean ISC of the activation time courses of individual subjects was 0.31 ± 0.03 (p < 0.001).

The time courses of IC1 and IC2 are very similar (i.e. they are as sensitive) when there is speech (pink bars in Figure 4) in the soundtrack. This raises the possibility that they reflect parallel aspects of speech processing such as acoustic *vs.* motor-cue based speech perception [23]. However, there are marked differences during music (blue bars), IC1 showing more prominent activation during music than IC2. While this tentatively suggests that IC2 is more selective to speech this difference is not strongly reflected in the weights of the stimulus features: music receives similar weights for both ICs in the linear fitting process, although singing receives a high weight only with IC1. This is seen in the time courses where IC1 activity increases during singing (yellow) compared to instrumental music (blue), but IC2 activity does not.

**Figure 5:**
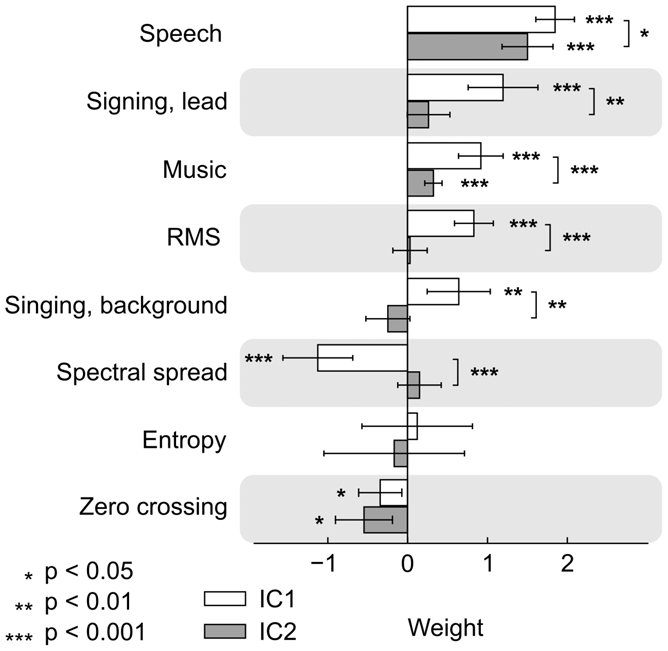
Beta weights of IC1 and IC2 in single-feature GLM analysis. Asterisks indicate when the weights differ signigicantly from zero, or from each other. Tuning of IC1 (positive weigths) is clearly more shallow than that of IC2.

To directly test whether IC2 is more selective to speech than IC1 we performed GLM analyses with single auditory features for both ICs. Figure 5 depicts the weights for both ICs in the single feature analyses. The weights for speech, lead singing, music, RMS energy and spectral spread differ significantly between the ICs. Both ICs are sensitive to speech and music, but IC1 is also sensitive (positive beta weights differ significantly from zero) to lead singing, RMS energy and background singing. The profile of the positive weight strengths indicates that tuning of IC2 is steeper, i.e. it is more selective to speech than IC1. Note that the normalized weight strengths in Figure 4 and weight strengths in Figure 5 are different. This is due to the features not being orthogonal to each other. The weights in Figure 4 represent the optimal weights in fitting the sum of categories to the observed activation whereas the weights in Figure 5 indicate the optimal coefficients for single features. Additionally, to facilitate the comparison, weights depicted in Figure 5 were not normalized. To further elucidate the activity differences in IC1 and IC2 we also performed voxel-wise analysis of the stimulus features separately (see below).

**Figure 6:**
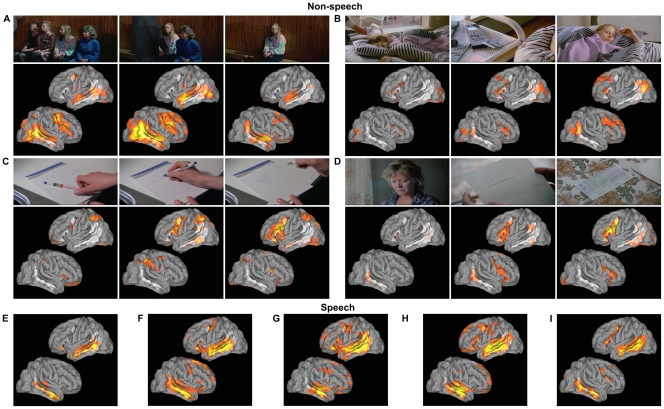
Brain activity patterns during scenes causing high activity in speech-sensitive IC2. Letters refer to those in Figure 4. Location of IC2 is indicated in white. Overlaid (red–yellow) is the mean standardized activity level across subjects. The activity is thresholded at p < 0.05, assuming the signal is the mean of ten random samples from standard normal distribution. Scene in A is associated with strongly right-lateralized activity in temporal and frontal areas and moderate activity in superior and posterior temporal lobe in the left hemisphere. Scene in B is associated with moderate activity in frontal areas and posterior temporal areas of IC2, and widespread activity in the right PFC. Scenes in C and D are associated with strong and wide spread activity in left ventral premotor cortex and some activity in the middle temporal and right premotor areas. Scene in C is also associated with high activity in parietal regions along the intraparietal sulcus. Panels E–I show typical activity patterns during scenes containing speech. Activity is seen particularly along STS/MTG and frontal areas with stronger and more widely spread activations in the left hemisphere. (Still images by courtesy of Aki Kaurismäki and Sputnik Oy).

The time course of IC2 shows four peaks (Figure 4: A, B, C, D) which are not explained by the annotated sound features, and five peaks (E, F, G, H, I) which coincide with speech. While the activity levels are similar during all of these peaks, the activation patterns within the IC2 differ (see Figure 6). Speech activates most of the areas included in IC2. Activity during peaks A–D is located in different parts of the IC and, critically, the left STS/MTG region shows little activity in scenes that do not contain speech.

#### Visual independent components

**Figure 7:**
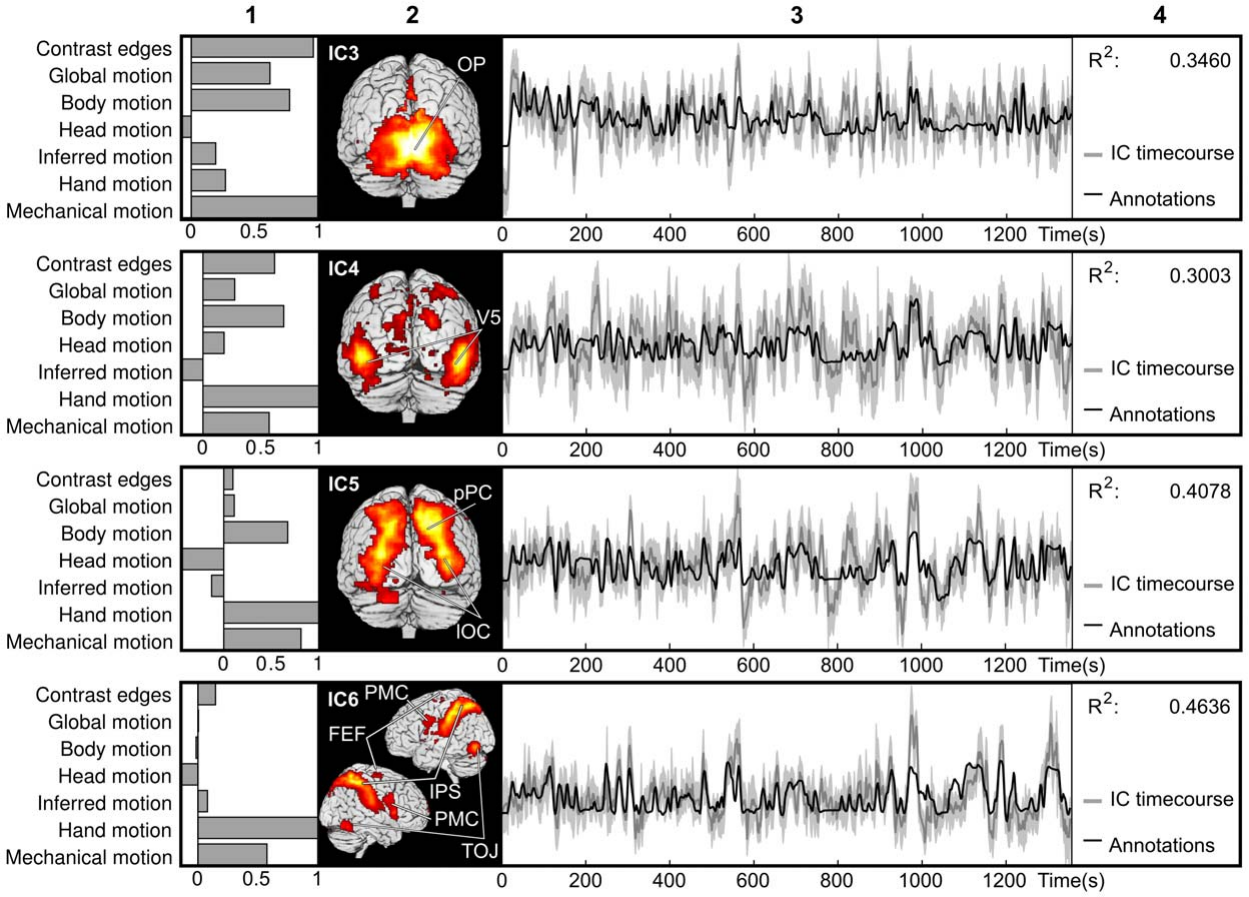
The four ICs that were sensitive to visual features in the movie. From left to right are shown: 1) the normalized weights used to fit the visual feature model to IC activity, 2) the activation patterns of the ICs plotted on the cortical surface, 3) mean IC time courses (dark gray), 95% confidence interval of the mean (light gray), and fitted annotation time courses (black), and 4) the coefficient of determination (R^2^) of the fitted model with the mean IC time course. IC3, located in occipital pole, received high weights to most visual categories, particularly to contrast edges and mechanical, global and body motion. IC4, located in posterior temporal lobe and lateral occipital lobe overlapping motion sensitive visual area V5 also received high weights for most visual categories, and especially for hand motions. IC5 is located in lateral occipital lobe and posterior parietal areas, and IC6 encompasses a network including intraparietal sulcus (IPS), frontal eye fields (FEF), ventral premotor cortex (PMC) and inferior temporo-occipital junction (TOJ). IC6 predominantly correlated with occurrence of hand and mechanical movements, whereas IC5 additionally showed preference to body motion.

Figure 7 depicts all ICs sensitive to various types of visual features. IC3 is located in the occipital pole (OP) and superior-posterior aspects of the cerebellum. IC3 follows the dynamics of contrast edges in the image, global motion, body motion and mechanical motion with R^2^  =  0.3460. However, it is only slightly sensitive to hand and inferred motions, and not at all to head motion. Mean ISC of the activation time courses of individual subjects was 0.36 ± 0.02 (p < 0.001).

IC4 coincides with the motion sensitive visual area V5 and extends to surrounding areas of the posterior temporal lobe and lateral occipital lobe. It shows highest sensitivity to hand motion (as also IC5 and IC6), but is also sensitive to other motion categories, except inferred motion. The stimulus model fit R^2^  =  0.3003, and mean ISC of the activation time courses of individual subjects was 0.25 ± 0.02 (p < 0.001).

IC5 includes posterior region of the parietal lobe and lateral-ventral aspects of the occipital lobe, and posterior lobe of the cerebellum on the left. Similarly to IC4 and IC6, IC5 is sensitive to hand and mechanical motion. It also shares sensitivity to body motion with IC3 and IC4. The stimulus model fit R^2^  =  0.4078, and mean ISC of the activation time courses of individual subjects was 0.36 ± 0.01 (p < 0.001).

IC6 is located in superior parietal lobule including intraparietal sulcus (IPS), inferior part of the temporo-occipital junction (TOJ), ventral parts of lateral premotor cortex, and the frontal eye fields (FEF). The visual stimulus model fits the activity with R^2^  =  0.4636. IC6 is very sensitive to hand motion, and to a lesser extent to mechanical motion. Mean ISC of the activation time courses of individual subjects was r  =  0.38 ± 0.03 (p < 0.001).

As is revealed by the weights of the annotations, sensitivities of these four visual-processing related ICs differ in their specificity to annotated visual features. IC3 and IC4, reflecting activity in lower-order visual areas, are sensitive to different types of visual motion and contrast edges. IC5 and IC6 are quite insensitive to contrast edges, but sensitive to visual motions. IC6 is most selective to a specific type of motion, hand movements, but also to mechanical motion.

Similar fitting as in Figure 7 was performed on the semi-automatically annotated motion to cross-validate the different approaches of quantifying visual motion. Because of similarity of the models, the results were very similar to those presented in Figure 7. The overall model fit was slightly lower for automatically extracted model of motion (R^2^ values: IC3  =  0.3097, IC4  =  0.2543, IC5  =  0.2855, IC6  =  0.307) but the relative weights were almost identical. Therefore, we show only results for the manually annotated motion model.

#### Voxel-wise analysis

In addition to the analysis where the time courses of the brain networks captured by the ICs were fitted to the time courses of stimulus annotations, we performed GLM fitting of the stimulus annotation time courses with individual-voxel time courses using both the across-subjects averaged voxel time courses as well as single-subject data. The overall model fit for single-subject data was lower (sound mean r  =  0.6418, motion mean r  =  0.5143 for maximum voxel) than for the mean activity across subjects (sound r  =  0.8493, motion r  =  0.7275 for maximum voxel) presumably due to lower signal-to-noise of the single-subject BOLD data. However, the areas of best fit were highly similar with these two approaches. Therefore, we present here only the results of the group-level analysis.

**Figure 8:**
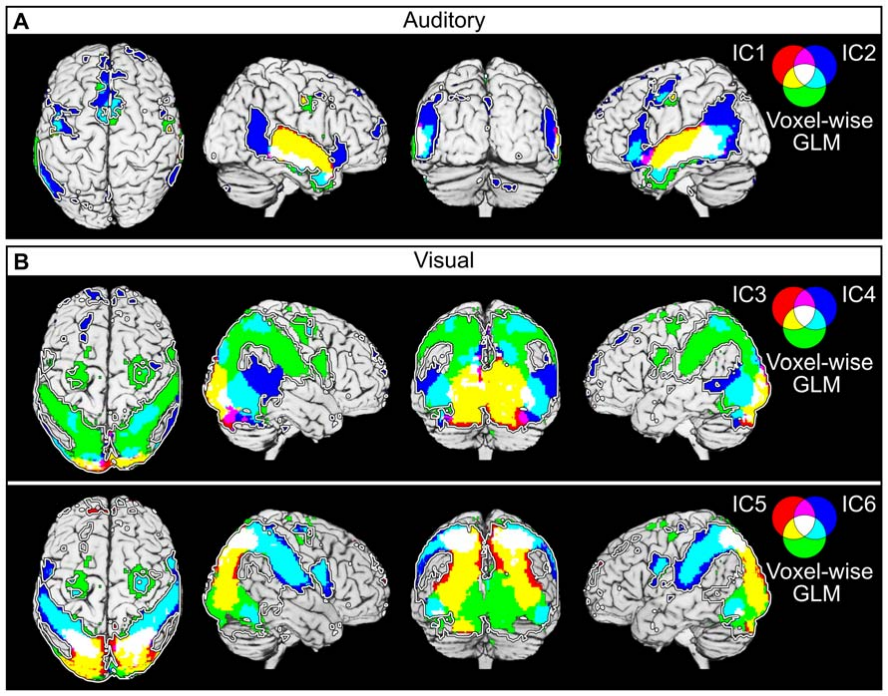
Comparison of the ICA and voxel-wise results. Voxel-wise results showing significant correlation (p < 0.001) with the auditory (A) and visual (B) stimulus model are shown in green and ICs in red and blue. Yellow and cyan indicate areas where voxel-wise results overlap with one of the ICs, and white indicates areas where the voxel-wise results overlap with both two ICs. Total area of cortical surface covered by the auditory (A) and visual ICs (B) are indicated by a white line on black background.

Figure 8 shows the IC and voxel-wise results overlaid with green color indicating areas that were seen only with the voxel-wise GLM, red and blue colors indicating areas disclosed by the respective IC components, and white, yellow, and cyan indicating areas of overlap between the IC components and GLM analysis, respectively. Figure 8 A shows the overlap between the two auditory ICs and the GLM model. GLM results cover largely the same area as IC1. However, IC2 extends to posterior temporal lobe regions that are not revealed by the voxel-wise GLM analysis. Furthermore, the dorsomedial prefrontal cluster of IC2 extends to anterior parts of the PFC beyond the SMA cluster revealed by voxel-wise analysis. Conversely, in left anterior MTG the voxel-wise results extend slightly beyond the sound-related ICs. Visual IC4 (Figure 8 B, top) extends to posterior regions of the temporal lobe that are not revealed by the voxel-wise GLM analysis, very much like the auditory IC2. As is shown in the following section, these differences are probably due to ICs reflecting the functional network structure (*i.e.,* there is a high correlation between brain areas contained in each IC), whereas the voxel-wise GLM based analysis reflects the relation of each individual voxel and the stimulus model. Because the thresholds for significant correlation were between r = 0.41 and r = 0.48 for both region-region and region-model correlations it is possible that the significant functional connectivity may be caused by variance in the data which is not explained by the model. Figure 8 also demonstrates how the IC-based analysis helps to functionally parcellate the areas that are revealed by the voxel-wise GLM analysis, without information of the stimulus features.

**Figure 9:**
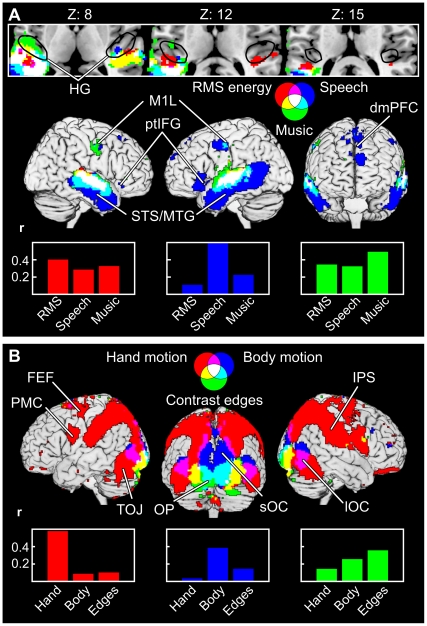
Areas showing significant correlations with single auditory and visual features. The color coding as in Figure 8. Results are thresholded at p < 0.001. A: Speech explains activity in the superior temporal sulcus (STS) and middle temporal gyrus (MTG), lip representation area of the primary motor cortex (M1L) and ptIFG particularly in the left hemisphere, and dorsomedial PFC. Partially overlapping areas also show activity correlated with music. RMS energy explains activity in the superior temporal areas, particularly in a part of the posterior bank of Heschl’s gyrus (in or in the vicinity of the primary auditory cortex) and/or Planum Temporale. Other sound categories are not significantly correlated with activity in this region. The black outline indicates the area encompassing the Heschl’s gyrus (HG) of all subjects visually identified from the standardized structural images. Bar graphs show the correlation coefficients for each stimulus feature in the non-overlapping areas. Colors of the bars refer to the brain areas best activated with one of the three stimulus features. B: Hand motion activates strongly IPS and TOJ. The areas are very similar to those included in IC6 (see Figure 7). Superior occipital cortex (sOC), occipital pole (OP), and parts of the lateral occipital cortex (lOC) show specific activity to body motion. Activity in the occipital pole also correlated with the contrast edges of the image.

Figure 9 depicts brain areas that had a strong correlation in the voxel-wise analysis with three auditory (Figure 9 A: RMS energy, speech, music) and visual (Figure 9 B: hand motion, body motion, contrast edges) features. Superior temporal areas are sensitive to all three auditory features (white) while the inferior temporal parts and ptIFG are correlated only with speech (blue). Notably, an area overlapping the location of posterior bank of Heschl’s gyrus (HG) and anterior aspects of planum temporale is significantly correlated only with the RMS energy (red) of the sound track but not with the other auditory features included in this analysis at the selected significance level. In addition to the features presented in Figure 9, parts of the superior temporal and motor cortex exhibited overlapping areas sensitive to both music and speech that were also correlated with lead singing, but we failed to see any areas outside the speech and music sensitive regions that would have correlated with instances of singing. Bar graphs show the correlation coefficients of the different stimulus models with mean time courses of those areas that were significantly sensitive to only one category. The bar colors match the areas in the brain images. The red areas are most correlated with RMS energy but also show relatively high correlation with music and speech. Blue areas show high correlation with speech, some correlation with music but relatively low correlation with RMS energy. Green areas correlate with music but also with speech and RMS energy.

As seen in Figure 9 B, hand motion is correlated with a set of brain areas (red) that resembles IC6 (Figure 7). Temporo-occipital parts of the hand motion sensitive areas are also correlated with body motion as indicated by purple color. Activity in superior occipital and posterior parietal areas (blue) and occipital pole (cyan) is sensitive to body motions while only occipital pole is sensitive to the contrast edges (green, cyan and yellow). In addition to contrast edges and body motions, mechanical motion category was also correlated with activity in the occipital pole while global motion category was correlated with activity in anterior parts of the medial occipital lobe. These results follow closely the regional specialization revealed by the weights of the stimulus categories in the ICs in Figure 7. Bar graphs show that red areas are very specific to hand motions compared to the other categories. Blue areas are most sensitive to body motions. Green areas show highest correlations with contrast edges, but correlations with other categories are also relatively high as was implied by the weights for the categories for IC3 (Figure 7).

#### Comparison of ICA and voxel-wise results

**Figure 10:**
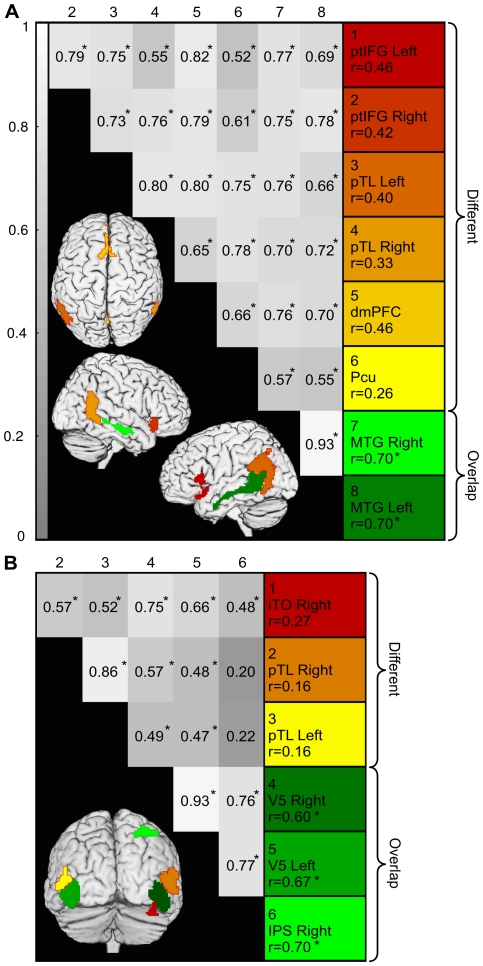
Comparison of overlapping *vs.* non-overlapping ROIs in ICA and voxel-wise GLM results. A: Comparison of speech sensitive IC2 and areas correlated with the auditory model in the voxel-wise analysis. LEFT: Areas where ICA and voxel-wise results differed are color-coded with red–yellow, and overlapping areas are green. Pair-wise correlation matrix of the mean time courses of each ROI is presented on grey background, where the brightness of the grey shade corresponds to the correlation coefficient. Asterisks indicate significant correlations. RIGHT: The correlation coefficients of the auditory model with each ROIs time courses. Color coding corresponds to the colors on the brain images. All ROIs correlated strongly with each other, but only the ROIs which were present in both ICA and voxel-wise results are significantly correlated with the auditory model. B: Comparison of motion sensitive IC4 with voxel-wise results. Details as in A; iTO refers to inferior temporo-occipital ROI. Non-overlapping ROIs are not significantly correlated with the stimulus model, but all ROIs are significantly correlated with the activity of the strongest clusters of IC4 centered on the area V5.

To assess the differences of results revealed by ICA and voxel-wise analyses in Figure 8 (IC2 and IC4), we isolated regions of interests (ROIs) showing differences between the ICs and voxel-wise results and compared them to ROIs in which results were similar. While IC2 and voxel-wise results overlapped in the lower bank of the STS/MTG (green in Figure 10 A), IC2 additionally extended to the ptIFG, posterior temporal lobe (pTL)/TPJ, dmPFC, and PCu (red to yellow in Figure 10 A). Small (<125 voxels) clusters in the IFG and PMC were omitted in the current analysis. IC4 and voxel-wise results overlapped in areas centered on the V5 bilaterally and in the right IPS (green in Figure 10 B). However, IC4 extended to pTL and inferior temporo-occipital regions (red to yellow in Figure 10 B).

We calculated the correlations of the mean time courses of the ROIs with each other and with the stimulus model (Figure 10). For both visual and auditory activations, the areas included in the ICs which do not overlap with voxel-wise results are significantly correlated with at least two ROIs which were included in both ICA and voxel-wise results. However, none of the non-overlapping areas correlated significantly with the stimulus models when using the thresholding based on the permutation test. Figure 6 additionally demonstrates that while occurrences of speech typically activate most of the region covered by IC2 (Figure 6 E–I), other stimulation causes independent activations only in isolated parts of the IC (Figure 6 A–D), which partially coincide with the ROIs not present in the voxel-wise results in Figure 10 A. These analyses demonstrate that the correlations of activity and stimulus model between different parts of an IC may differ. Moreover, functional areas included in one IC need not be constantly acting in unison, but may be involved in multiple network configurations changing in time, and activity in only part of the network may cause significant activity peaks in the time course of the IC.

## Discussion

Our results demonstrate the feasibility of using highly complex naturalistic stimuli such as feature films in neuroimaging studies to disclose how the human brain processes information under naturalistic conditions approaching those that one encounters in real life. Previous neuroimaging studies using movies as stimuli have demonstrated that brains of individual subjects react similarly [4], [5], and that ICA [3], [5] and linear modeling of stimulus features [10] can be used to obtain physiologically feasible activation patterns during watching of movies. Here, we demonstrate how detailed annotations of stimulus features can be combined with ICA and voxel-wise analysis approaches to help tease apart the specific roles that each brain area and functional network play when one perceives a rich and dynamic naturalistic audiovisual stimulus. We further highlight important differences in the two approaches that one should consider when analyzing data collected in naturalistic experiments and show that when results are calculated over the whole dataset neither method catches the fine grained differences between the spatial activity patterns observed in shorter time windows during the movie.

Auditory ICs depicted in Figure 4 segregated brain areas related to low-level auditory features (IC1) and higher level features (IC2), the latter being particularly selective for speech but also responding to music (see Figure 5). IC2 additionally activated during scenes of non-verbal communication (being the only one left without a dance partner, leaving money on the night table after a sexual encounter, writing, and reading), however, the detailed pattern of activation differed strongly compared to scenes containing speech (see Figure 6). Particularly the left ptIFG activating in Figure 6 B–D, but also the right ptIFG in Figure 6 A have been previously associated with understanding and observation of actions [24] among other functions. These differences suggest that while ICA may group brain areas to be a single IC when the analysis is based on the whole dataset, some sub-areas of the ICs may still exhibit different activity patterns when shorter time periods are analyzed.

Analogously to the auditory case, our results, obtained by combining stimulus annotations with ICA and voxel-wise analysis (see Figure 7 and Figure 9 B) functionally delineate visual areas according to their sensitivity to visual features. Early areas in the occipital lobe are modulated by a large variety of visual features, particularly the contrast edges in the image, and moving higher along the dorsal stream the areas become more specialized. Particularly, IPS together with FEF and ventral premotor cortex was found to be almost exclusively sensitive to hand motions. Sensitivity of the IPS to manual manipulations as well as using tools to manipulate objects has been suggested using traditional neuroimaging paradigms particularly in the rostral regions of IPS together with activity in the premotor cortex [25]. Activity of parietal and premotor areas is often attributed to the mirror neuron system (for a review, [26]) and somatotopic organization of the IPS [27] has also been reported, demonstrating rostral regions of the IPS to be sensitive to hand and lip movements while posterior parietal areas, partially overlapping the body sensitive areas in our study, are sensitive to foot movements. Others have proposed the IPS to be more related to directing of visual and auditory attention [28], [29], but these two suggestions need not be mutually exclusive.

Spatial ICA extracts independent networks of brain activity revealing functionally connected brain areas [30]. If various stimulus features occur simultaneously, ICA may group the specific areas responsible for encoding these features into a single IC. This is also true for model-based analysis methods. However, during a long feature film, any two given stimulus features rarely co-occur entirely, and indeed as is seen in Figure 7, the three highly correlated and partially overlapping networks activating consistently to motion (IC4, IC5, and IC6) suggest that ICA may be useful in separating networks, which model-based methods may not be able to separate. While the peak timings of all three ICs are fairly similar, the peak amplitudes differ. As we demonstrated, in this case similar separation can be achieved by careful modeling of the stimulus. However, one of the advantages of ICA is that it can be used to find areas with similar functional connectivity across subjects that need not be directly related to the physical features of the stimulus. In fact, when performing group ICA on temporally concatenated data the ICs are not required to activate similarly across subjects. This is especially important given high inter-individual variability in temporal dynamics of functional activity related to higher cognitive processes [31] and, thus, to extract such activity patterns using model-based methods one would need separate models for each subject. However, this problem is not removed by the use of ICA alone as it does not reveal the functional significance of the network. As an example, the fit of the sensory models used in the present study decreased as the temporal correlation across subject pairs decreased. Obviously, the more different the activity of functional networks of individual subjects is, the higher is the need for individual models. The present results demonstrate that ICA is a powerful method to complement model based approaches, but connecting temporal activation of ICs to specific stimulus features and stages of information processing always requires careful modeling of the stimulus and subject’s behavior.

Temporal lobe and lip representation area of the motor cortex, and inferior frontal gyrus pars triangularis encompassed by IC2 were found to be sensitive to speech. These areas are very similar to a widespread network of temporal and frontal areas linked to processing of natural connected speech over long timescales, but not to processing of scrambled sentences [32]. Primary motor areas that were activated during speech were also activated by music and singing, which is in line with prior research showing similar areas activating during speech, music and singing perception as well as music discrimination [33], [34]. A distinct representation of other sound features was apparent only in early auditory areas of the superior temporal lobe. Particularly, a region in the posterior bank of Heschl’s gyrus and anterior aspect of planum temporale was the only brain area that was correlated with the RMS energy of the soundtrack but not with other auditory features. These areas overlap with the presumed location of the primary auditory cortex (i.e., the medial two-thirds of HG), and the result is consistent with findings of prior research using simplified paradigms [35] reporting loudness-sensitive responses in the Heschl’s gyrus. However, it should be noted that despite not being significantly correlated with the activity in the Heschl’s gyrus at the current threshold, music still correlated relatively strongly with the same areas, which is expected as the scenes containing music tended to be relatively loud. Despite the correlated activity in superior temporal areas RMS energy did not receive high weights when annotations were fitted to the IC time courses. When annotations were considered alone the activity in the area of IC1 was better explained by RMS energy than sound entropy (see Figure 5), which was not significantly correlated with auditory cortex activity even though it received a relatively high weight in the fitting process. It is, therefore, important to confirm the sensitivity implied by the weights of the GLM with other measures when the features of the model are correlated.

ICA was not sensitive enough to reveal presumed fine-grained differences in sensitivity to different acoustic features in the auditory cortical areas. Therefore, it was valuable to complement the data driven analysis with model-based methods. Evidently, there are situations where a well-defined model may be able to separate functionally distinct regions, when they are not independent enough to be segregated with blind source separation techniques. Depending on the amount and quality of data and the employed implementation ICA methods may be used to extract a higher number of components than was achievable in the current study presumably helping to capture many of the distinctions between cortical areas we have demonstrated using model-based methods. However, the stability of source estimates should be analyzed to evaluate their reliability as increasing the number of ICs may lead to over fitting and unstable IC estimates. The selection of the number of ICs to be estimated is typically rather arbitrary and the methods used for estimating the appropriate number of components often yield relatively high estimates leading to unstable ICs. While using multiple methods in the analysis of any data without appropriate corrections for multiple post-hoc comparisons does increase the risk for false positive findings, a voxel-wise analysis is a worthwhile “sanity check” for functional connectivity analyses (such as ICA) when it is possible to conduct.

Reverse correlation methods have been suggested for finding the stimulus features related to the observed activity of brain areas during natural viewing of complex stimuli [4], [36]. Bartels and Zeki [3] noted that such approaches are susceptible to false interpretations because the method leaves a lot of room for human interpretation. They suggested a two-step process of quantification of the features where first the IC time courses are used to inform the selection of features to be annotated, and these features are then quantified and used for analysis. However, hypotheses derived from the fMRI data cannot be tested with the same dataset without biasing the outcome. Therefore, the parameters to be modeled should be independently defined prior to analysis. Despite this caveat, dimension reduction through ICA makes the postulation of novel data-driven hypotheses for subsequent experiments feasible, thus making the reverse correlation approach a useful addition to predefined modeling of the data as long as the data that the hypotheses are tested on is independent from the data that is used to generate the hypotheses.

We have taken the exploration of data one step further by looking at the time dependent activity levels of voxels over the whole brain synchronized with the movie stimulus (Figure 6). To understand what happens in the brain during complex stimulation it is important to carefully analyze the time-dependent activity of individual brain areas. Neither ICA nor voxel-wise GLM analysis over long time scales uncover the moment to moment differences between the sub-regions of the areas they reveal. However, because naturalistic stimulation causes highly replicable activity patterns across subjects the visualization of average activity of a group requires relatively low number of subjects without producing an excessive number of false positive activations. This allows the examination of spatial activation patterns at each time point. As an example of this, the observation that parts of the speech sensitive network of brain areas of IC2 also activated during non-vocal communication may warrant further studies.

While the results of the current study demonstrate that it is possible to extract simple stimulus features and find their neural correlates in a naturalistic context, selecting and modeling the relevant features for the model is not trivial. Manual annotation is laborious, which makes it important to develop accurate algorithms to annotate both optic flow and acoustics of the complex stimuli as accurately as possible. However, at least at present, these annotations need to be checked by the experimenter. Furthermore, as suggested by our results, the brain activity is not likely to be linearly dependent on the raw stimulus properties and manual annotations of, in this case, perceptual motion strength may better fit the brain activity than automated ones. In addition, many important aspects of the movie are missed if only the sensory properties are modeled. Therefore, expert annotations, subjective ratings, and behavioral data may play a key role in finding neural basis for endogenous/higher order cognitive and emotional processes not directly related to physical qualities of the stimulus. Further work on exploring what combinations of sensory features and endogenous processes best explain the observed brain activity is of great importance in trying to form a cohesive theory of how the human brain processes the complex natural world.

### Conclusion

Our results suggest that patterns of brain activity underlying the processing of various acoustic and visual features can be effectively studied using highly complex audiovisual stimulation, such as a feature film, by combining detailed annotations of the stimulus with ICA and voxel-wise analyses, and even unguided exploration of whole brain fMRI data. We have further demonstrated important differences between the employed methods. Studying how the human brain processes information under naturalistic conditions is an important addition to the repertoire of paradigms aiming to increase our understanding of the processing of stimulus features in the human brain. Naturalistic experiments have the potential to reveal such aspects of processing of especially complex features which are difficult or even impossible to discover using very simple stimuli and tasks. Further, the approach we present here could be extended in future studies with self-reports of subjective experiences such as emotional states as well as complementary behavioral measures such as tracking of eye-movements to enable more comprehensive investigation of the neural basis of human perceptual, cognitive and emotional processes.
